# Functional connectivity of the human amygdala in health and in depression

**DOI:** 10.1093/scan/nsy032

**Published:** 2018-05-14

**Authors:** Wei Cheng, Edmund T Rolls, Jiang Qiu, Xiongfei Xie, Wujun Lyu, Yu Li, Chu-Chung Huang, Albert C Yang, Shih-Jen Tsai, Fajin Lyu, Kaixiang Zhuang, Ching-Po Lin, Peng Xie, Jianfeng Feng

**Affiliations:** 1 Institute of Science and Technology for Brain-Inspired Intelligence Fudan University, Shanghai 200433, China; 2 Department of Computer Science, University of Warwick, Coventry CV4 7AL, UK; 3 Oxford Centre for Computational Neuroscience, Oxford, UK; 4 Key Laboratory of Cognition and Personality (SWU), Ministry of Education, Chongqing, China; 5 Department of Psychology, Southwest University, Chongqing, China; 6 Department of Radiology, The First Affiliated Hospital of Chongqing Medical University, Chongqing, China; 7 School of Mathematics, Shanghai University Finance and Economics, Shanghai 200433, PR China; 8 Institute of Neuroscience, National Yang-Ming University, Taipei, Taiwan; 9 Department of Psychiatry, Taipei Veterans General Hospital, Taipei, Taiwan; 10 Brain Research Center, National Yang-Ming University, Taipei, Taiwan; 11 Institute of Neuroscience, Chongqing Medical University, Chongqing, China; 12 Chongqing Key Laboratory of Neurobiology, Chongqing, China; 13 Department of Neurology, The First Affiliated Hospital of Chongqing Medical University, Chongqing, China; 14 School of Mathematical Sciences, School of Life Science and the Collaborative Innovation Center for Brain Science, Fudan University, Shanghai 200433, PR China

**Keywords:** depression, amygdala, orbitofrontal cortex, functional connectivity, emotion

## Abstract

To analyse the functioning of the amygdala in depression, we performed the first voxel-level resting state functional-connectivity neuroimaging analysis of depression of voxels in the amygdala with all other voxels in the brain, with 336 patients with major depressive disorder and 350 controls. Amygdala voxels had decreased functional connectivity (FC) with the orbitofrontal cortex, temporal lobe areas, including the temporal pole, inferior temporal gyrus and the parahippocampal gyrus. The reductions in the strengths of the FC of the amygdala voxels with the medial orbitofrontal cortex and temporal lobe voxels were correlated with increases in the Beck Depression Inventory score and in the duration of illness measures of depression. Parcellation analysis in 350 healthy controls based on voxel-level FC showed that the basal division of the amygdala has high FC with medial orbitofrontal cortex areas, and the dorsolateral amygdala has strong FC with the lateral orbitofrontal cortex and related ventral parts of the inferior frontal gyrus. In depression, the basal amygdala division had especially reduced FC with the medial orbitofrontal cortex, which is involved in reward; and the dorsolateral amygdala subdivision had relatively reduced FC with the lateral orbitofrontal cortex, which is involved in non-reward.

## Introduction 

There is considerable evidence that the amygdala is involved in emotion (Aggleton, 2000; Whalen and Phelps, 2009; LeDoux, 2012; Rolls, 2014, 2018). Moreover, resting state functional connectivity (FC) between brain areas, which reflects correlations of activity, is a fundamental tool in helping to understand the brain regions with altered connectivity and function in mental disorders (Deco and Kringelbach, 2014), and changes in amygdala FC have been related to depression (Disner *et al.*, 2011; Phillips *et al.*, 2015; Loonen and Ivanova, 2016; Vai *et al.*, 2016; Connolly *et al.*, 2017; Straub *et al.*, 2017). Furthermore, the amygdala responses to faces (Leonard *et al.*, 1985) may be influenced by antidepressant drugs (McCabe *et al.*, 2012; Harmer and Cowen, 2013).

The aim of the present paper was to examine the FC of the amygdala in depression at the voxel level. We analysed every amygdala voxel for significantly different FC with voxels throughout the rest of the brain in depressed people *vs* controls, and tested whether any differences of FC found are correlated with the symptoms of depression, in order to advance understanding of the amygdala and depression. The advantage of voxel-level FC is that we can show exactly which amygdala voxels have altered FC with the exact (voxel-level) parts of other brain areas. In order to perform this voxel-level FC analysis, we utilized and required a uniquely large sample of 336 patients with major depressive disorder and 350 controls. In a previous study with 70 participants, lower FC between the amygdala and hippocampus and parahippocampus was reported (Cullen *et al.*, 2014).

This investigation is very different from a previous analysis of FC in depression (Cheng *et al.*, 2016), as follows. First, we focused on the FC of the amygdala, not the whole brain, in order to analyse the FC of the amygdala in depression. The methodology used here is quite different from, and more statistically powerful than, a whole brain voxel-to voxel FC analysis, which, because there are so many voxels pairs in the whole brain, is rather insensitive, for it carries a huge burden to correct for the multiple comparisons [1 133 760 771 voxel pairs requiring normally *P* < 10^−8^ for any effect to be significant (Cheng *et al.*, 2016)]. The patients in the present study included some of those in our earlier study for whom we had the data needed for the present study. Second, we performed a parcellation of the amygdala based on its FC, showed which parts the brain each amygdala subdivision was related to, and showed how the FC of each amygdala subdivision was different in depression. Third, we describe here how amygdala connectivity was correlated with the depression severity and duration, which was not performed in the previous study. Part of the reason for these differences is that in the previous investigation we focused on voxel-to-voxel whole brain connectivity, which limits the results that can be established, whereas here we focus on the amygdala, and are able to report significant differences in its FC in depression, and even of its subdivisions.

A new theory of depression is that the lateral orbitofrontal cortex has increased sensitivity of a non-reward attractor in depression, and that the reciprocally related medial orbitofrontal cortex reward system is underactive in depression (Rolls, 2016c), and there is evidence consistent with this (Eshel and Roiser, 2010; Russo and Nestler, 2013; Whitton *et al.*, 2015; Cheng *et al.*, 2016; Rolls, 2016c; Rolls *et al.*, 2017; Cheng *et al.*, 2018a,b). It was therefore of interest in the present investigation whether the amygdala had altered connectivity with these other brain systems already implicated in depression. The new theory is that the lateral orbitofrontal cortex, which is activated when expected reward is not obtained (which can cause sadness), can enter and maintain an ongoing mood state in a recurrent ‘attractor’ network more readily in depression (Rolls, 2016c), as described more fully in the ‘Discussion’ section.

## Methods

### Participants

There were 336 patients with a diagnosis of major depression and 350 controls. The data available for this study were from Xinan (First Affiliated Hospital of Chongqing Medical School in Chongqing, China) and Taiwan (Veteran General Hospital, Taipei). This is a subset of participants in a previous study in which very different analyses were performed (Cheng *et al.*, 2016), as is made clear in the Introduction. All participants were diagnosed according to the Diagnostic and Statistical Manual of Mental Disorder-IV criteria for major depressive disorder. Depression severity and symptomatology were evaluated by the Hamilton Depression Rating Scale (HAMD, 17 items) (Hamilton, 1960) and the Beck Depression Inventory (BDI) (Beck and Beamesderfer, 1974). One hundred and twenty-five of the patients were not receiving medication at the time of the neuroimaging. Table 1 provides a summary of the demographic information and the psychiatric diagnosis of the participants, with further details in the Supplementary Material.

**Table 1.** A summary of the demographic information and the psychiatric diagnosis in the present study

Sites	Group	Age (years)	Sex (male/female)	Education (years)	Medication (yes/no)	HAMD	BDI	Duration of illness	First episode(yes/no)	Mean FD
**Taiwan**	Healthy	49.18±8.58	60/36	15.04±3.83	–	–	–	–	–	0.133±0.054
	Patient	52.64±14.86	33/21	12.66±3.95	54**/**0	9.34±6.99	/	8.63±6.92	0**/**54	0.116 ±0.056
	Statistic (*t*/*P*)	−1.810**/**0.072	0.028/0.866	3.60**/**4.3e−4		–	–	–	–	1.833**/**0.0687
**Xinan**	Healthy	39.65±15.80	166**/**88	13.01±3.89	–	–	–	–	–	0.133 ±0.063
	Patient	38.74±13.65	183**/**99	11.91±3.58	157**/**125	20.8±5.87	20.42±9.33	4.16±5.51	209**/**49	0.125±0.054
	Statistic (*t*/*P*)	0.719**/**0.472	0.013**/**0.911	3.41**/**6.9e−4	–	–	–	–	–	1.729**/**0.084

Age, education, HAMD, BDI, duration of illness and Mean FD are presented in mean ± s.d.

HAMD, Hamilton Depression Rating Scale; BDI, Beck Depression Inventory; Mean FD = mean framewise displacements.

### Image acquisition and preprocessing

Data for resting state FC analysis were collected in 3T MRI scanners in an 8 min period in which the participants were awake in the scanner not performing a task using standard protocols described in the Supplementary Material.

Data preprocessing was performed using DPARSF (Chao-Gan and Yu-Feng, 2010) (http://restfmri.net) that is a toolbox based on the SPM8 software package. The first 10 EPI scans were discarded to suppress equilibration effects. The remaining scans of each subject underwent slice timing correction by sinc interpolating volume slices, motion correction for volume to volume displacement, spatial normalization to standard Montreal Neurological Institute (MNI) space using affine transformation and nonlinear deformation with a voxel size of 3 × 3  ×3 mm, followed by spatial smoothing (8 mm Full Width Half Maximum FWHM). To remove the sources of spurious correlations present in resting-state BOLD data, all fMRI time-series underwent band-pass temporal filtering (0.01–0.1 Hz), nuisance signal removal from the ventricles, and deep white matter, and regressing out any effects of head motion using the Friston *et al.* 24 head motion parameters procedure (Friston *et al.*, 1996). Finally, we implemented additional careful volume censoring (scrubbing) movement correction as reported by Power *et al.* (2014) to ensure that head-motion artefacts are not driving observed effects. The mean framewise displacement (FD) was computed with FD threshold for displacement being 0.5 mm. In addition to the frame corresponding to the displaced time point, 1 preceding and 2 succeeding time points were also deleted to reduce the spill-over effect of head movements. Subjects with >10% displaced frames flagged were completely excluded from the analysis as it is likely that such high-level of movement would have had an influence on several volumes. Global signals were not regressed out, for reasons described elsewhere (Cheng *et al.*, 2016). Because we do not regress out the global signal, most of the functional connectivities are positive, and the interpretation of a decrease of FC is just that the correlation between the activities of the pair of nodes has decreased. Considering the potential effect of gender (Tomasi and Volkow, 2012), age (Geerligs *et al.*, 2015) and head motion (Power *et al.*, 2012, 2014) on FC, any effects of gender ratio, years of education, age and head motion between the patient and control groups were regressed out in all analyses. There were no differences in the gender ratios, age and mean FD (*P* > 0.05 in all cases), though the number of years of education was lower in the patients than controls. However, none of the FC link differences found between patients and controls was correlated significantly (FDR, *P* < 0.05) with the number of years of education. We also note that the Taiwanese sample included patients with depression in remission while under antidepressant treatment, and thus their scores on the HAMD assessment were in the low range.

### Hypothesis based voxel-wise association studies

In this paper we utilize what we term ‘hypothesis-based voxel-level FC analysis’ in which we select a brain region of interest, but then calculate for every voxel in that region whether it has FC with individual voxels in every other brain region. In the present paper, we select the amygdala as the region of interest, given the research on it described above implicating it in depression, and then we show exactly which amygdala voxels have altered FC in depression with which individual voxels in every other brain area. Given that the amygdala has 149 voxels, and that there are 47 619 3 × 3 × 3 mm voxels in the automated anatomical atlas (AAL2) brain (Rolls *et al.*, 2015), the number of voxel pairs in this study was approximately (149 × 47 619). As noted in the section ‘Introduction’, this methodology is quite different from, and more statistically powerful than, a whole brain voxel-to voxel FC analysis, which, because there are so many voxels pairs in the whole brain, is rather insensitive, for it carries a huge burden to correct for the multiple comparisons [1 133 760 771 voxel pairs requiring normally *P* < 10^−8^ for any effect to be significant (Cheng *et al.*, 2016)].

In the present study, each resting-state fMRI volume included 47,619 voxels, and the amygdala region of interest had 149 voxels in the AAL2 atlas (Rolls *et al.*, 2015). For each pair of voxels in the amygdala and voxels in all other brain areas, the time series were extracted and their Pearson correlation was calculated for each subject, to provide the measure of FC, followed by *z*-transformation. Two-tailed, two-sample *t*-tests were performed on Fisher’s *z*-transformed correlation coefficients to identify significantly altered FC links in patients with depression compared with controls within each imaging centre. The effects of age, gender ratios, head motion (mean FDs) and education were regressed out within each dataset in this step by a generalized linear model (Barnes *et al.*, 2010; Di Martino *et al.*, 2014). After obtaining the *t*-test results (*P* value for each FC) for each centre, the Liptak-Stouffer *z* score method (Liptak, 1958) [described in detail in previous studies (Cheng *et al.*, 2015a,b, 2016)] was then used to combine the results from the individual datasets. Specifically, the *P*-value of each FC resulting from the two-sample *t*-test in the previous step was converted to its corresponding z score. This was calculated firstly as in equation: $z=\Phi^{-1}(1-p_{k})$, where Φ is the standard normal cumulative distribution function and k represents the k centre. Next, a combined *z* score for a FC was calculated using the Liptak-Stouffer formula: $Z=\sum w_{k}z_{k}/\sum w_{k}^{2}$, where $w_{k}=$ square root of sample size is the weight of the kth dataset. Finally, the *Z* is transformed into its corresponding *P*-value, and an FDR procedure was used to correct for multiple comparisons across the 149 × 47 619 voxel pairs. In the present study, FDR correction for the FC between any pair of voxels was used, and results are presented based on this statistical test with FDR *P* < 0.05, corresponding to a *P* threshold of $6.89\times 10^{-4}$ in the *Z*-tests.

### Visualization of the differences in FC for each voxel

To illustrate in some of the figures the extent to which voxels in different brain areas had differences of FC between patients and controls, we used a measure for the association (MA) between a voxel i and the brain disorder. This was defined as: $MA=N_{\alpha }$, where N is the number of links between voxel i and every other voxel in the brain that have a *P*-value of less than α (which in the present study with FDR correction was *P* < 0.05, corresponding to a *P* threshold of $6.89\times 10^{-4}$) in *t*-tests comparing patients with controls. A larger value of MA implies a more significant difference in FC. To ensure clarity, for a given voxel, the MA is the number of altered FC after FDR correction involving this voxel, so a higher MA indicates more significantly different FC links to other brain areas for that voxel.

### Clinical correlates

We also investigated whether the differences in FC between patients and controls were correlated with clinical variables [the HAMD (Hamilton, 1960), BDI (Beck and Beamesderfer, 1974), and illness duration (Bell-McGinty *et al.*, 2002; de Diego-Adelino *et al.*, 2014)]. Since the Taiwan dataset had only 54 patients and their HAMD scores are low due to the effects of medication, this correlation analysis was performed on the Xinan dataset. The samples that were more than 3 standard deviations away from the sample's mean were removed from this analysis. Specifically, for each brain region identified in the hypothesis based voxel-wise association studies, we first calculated the partial correlation between the clinical scores and the voxel-wise FCs between the significant voxels in that brain region and the amygdala, with head motion, education, sex and age as covariates so that they did not contribute to the correlation. Then the mean correlation between the clinical scores and voxel-wise FCs was defined as the overall correlation between the significant voxels in that brain region and the amygdala. Finally, a permutation test with 5000 randomizations of the patient labels was used to assess the statistical significance of the mean correlation.

## Results

The fMRI resting state FC analyses were performed with 336 patients with a diagnosis of major depression, and 350 controls, and this large population was sufficient to allow voxel-level analysis with FDR corrected statistics of the differences of FC of amygdala voxels with all other voxels in the brain (excluding the cerebellum) in patients *vs* controls.

### A voxel-level Association Study (vAS) of amygdala voxels with different FC in depressed patients

**Fig. 1.**
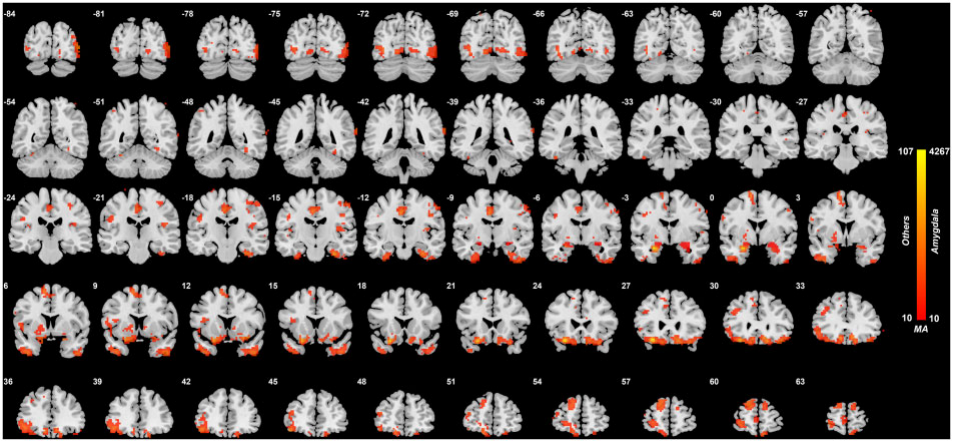
Anatomical location of consistently different FC in depression obtained from the voxel-based Association Study (vAS). Voxels showing the largest number of voxel-level FC differences with the amygdala in patients with depression. The colour bar represents the measure of association (MA) given by the number of significantly different FC links relating to each voxel. Voxels with MA larger than 10 are indicated here and elsewhere to show where the main differences are between patients with depression and controls. The right of the brain is on the right of each slice. The *Y* values are in MNI coordinates.

**Fig. 2.**
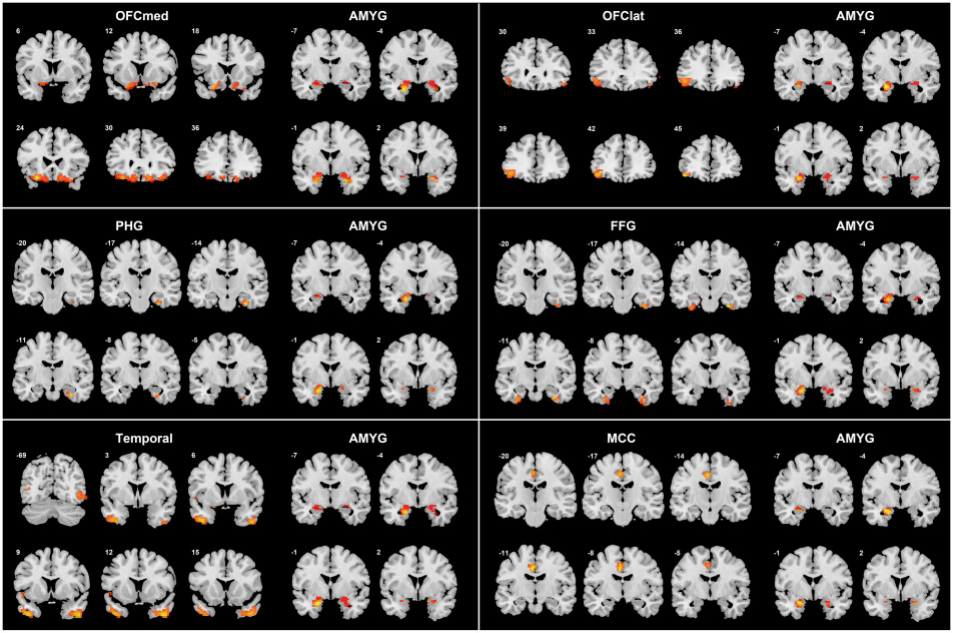
The voxel-level FC for amygdala voxels that are significantly different in the depressed and the control group, separated by the AAL2 region in which the significant voxels were located. For each AAL2 area illustrated, the left six slices through that area at the MNI Y level indicated show the locations of the voxels with different FC with the amygdala. The right four slices at *Y*=−2, 1, 4 and 7 show the amygdala voxels with different FC in depressed patients compared to controls for that brain area. MA values are shown. Voxels with decreased FC are shown in blue, and with increased FC in red/yellow. Voxels are indicated where the FC with the paired region is *P* < 0.05 (FDR corrected). For the Medial OFC (orbitofrontal cortex) subdiagram the AAL2 areas included were OFCmed, OFCant, OFCpost, Rectus and OLF (see Table 2). For the Lateral OFC the AAL2 areas included were OFClat and IFG_Orb. For the Temporal lobe the AAL2 areas included were TPO, ITG and MTG. These AAL2 areas (Rolls *et al.*, 2015) are listed in Supplementary Table S1.

As shown in Figures 1 and 2 and Table 2, there were a number of amygdala voxels with different FC in patients with depression compared to controls. In all cases, a reduction in FC was found in the depression group.

**Table 2.** Numbers of voxels in different AAL2 areas with significantly different FC with amygdala voxels in patients with depression

Areas	# Voxels	Peak *MA* value	MNI coordinates (Peak)		
			x	y	z
Frontal_Med_Orb_L, Frontal_Med_Orb_R, Rectus_L, Rectus_R, OFCmed_L, OFCmed_R, OFCant_L, OFCant_R, OFCpost_L, OFCpost_R, Olfactory_L, Olfactory_R	634	101	−30	27	−18
Frontal_Inf_Orb_2_L, Frontal_Inf_Orb_2_R, OFClat_L, OFClat_R	131	62	−24	12	−21
Hippocampus_R, ParaHippocampal_L, ParaHippocampal_R	54	60	30	−15	−30
Temporal_Pole_Sup_L, Temporal_Pole_Sup_R, Temporal_Pole_Mid_L, Temporal_Pole_Mid_R	198	47	45	9	−42
Fusiform_L, Fusiform_R, Temporal_Sup_L, Temporal_Sup_R, Temporal_Mid_L, Temporal_Mid_R, Temporal_Inf_L, Temporal_Inf_R	415	55	30	−15	−33
Cingulate_Mid_L, Cingulate_Mid_R	110	51	12	27	−21
Precentral_L, Precentral_R, Rolandic_Oper_L, Rolandic_Oper_R, Postcentral_L, Postcentral_R	199	34	−36	9	−42
Caudate_L, Caudate_R, Putamen_L, Putamen_R, Pallidum_L, Pallidum_R	81	23	−39	0	−42
Frontal_Sup_2_L, Frontal_Sup_2_R, Frontal_Mid_2_L, Frontal_Mid_2_R, Frontal_Inf_Tri_L	380	45	33	−18	−30
Insula_L, Insula_R	68	82	−27	21	−21
Cuneus_R, L ingual_L, Lingual_R, Occipital_Sup_L, Occipital_Sup_R, Occipital_Mid_L, Occipital_Mid_R, Occipital_Inf_L, Occipital_Inf_R	651	77	33	−9	−12
Frontal_Sup_2_L, Frontal_Sup_2_R	221	45	33	−18	−30
Amygdala_L, Amygdala_R	149	4267	−27	−3	−21
Frontal_Inf_Oper_L	41	24	−36	12	15
Frontal_Sup_Medial_L	26	15	−6	63	0
Calcarine_R	41	33	15	−93	12
Frontal_Sup_Medial_L, Frontal_Sup_Medial_R	58	39	−12	60	27
Supp_Motor_Area_L, Supp_Motor_Area_R	86	52	−12	0	69

The peak MA value shown is the number of links between the peak voxel in the brain regions listed under ‘Areas’ and the amygdala voxels, where these links are significantly different FDR corrected at *P* < 0.05. The MNI coordinates are the peak of the cluster in the brain regions listed under ‘Areas’. In all cases in this table, the FC links are weaker in the depressed group; thus these MA values show the number of decreased FC links in the depression group. There were a total of 97 740 links between the voxels of amygdala and other brain areas which showed a significant difference between controls and patients with depression (FDR correction, *P* < 0.05).

The largest clusters of voxels with altered (reduced) FC with the amygdala were in the medial orbitofrontal cortex (634 voxels, Table 2). (These voxel numbers are those with altered FC with amygdala voxels with *P* < 0.05 FDR corrected.) Additional areas with voxels with reduced FC with the amygdala in depression included the lateral orbitofrontal cortex, parahippocampal and fusiform gyri; inferior and middle temporal gyri; the temporal pole; the insula; occipital visual areas; the mid-cingulate cortex; the striatum (including parts of the caudate and putamen); and the precentral and postcentral gyrus (Table 2; Figures 1 and 2).

Supplementary Figure S3 confirms that the results with the combined dataset shown in Figure 1 are consistent with those obtained in a single dataset. Supplementary Figure S4 shows the correlation between the mean *t* value corresponding to the Xinan dataset and the mean *t* value corresponding to the Taiwan dataset for all the voxel-wise functional connectivities involving the amygdala. This provides confirmation that the results from the two datasets are consistent.

### Analysis of the FC links that were different in patients with depression

To investigate the brain areas between which there was different FC in depression, and whether it was increased or decreased, the FC of the voxels with significant differences of FC (after FDR correction at *P* < 0.05, and within the voxel clusters shown in Table 2) were measured for each of the AAL2 regions within which the voxels were located. (A list of abbreviations of the AAL2 areas are provided in Supplementary Table S1.) The FC differences are shown in Figure 2 at the voxel level, with the voxels shown arranged by the AAL2 areas in which they are found. Figure 2 shows that the amygdala voxels with altered FC with other brain areas tend to be in different parts of the amygdala. This is a new level of precision attained in this investigation in which the location in the amygdala of voxels with functional connectivities with the location of voxels in other brain areas are revealed, and how these differ in depression.

First, the different FC of amygdala voxels with the medial orbitofrontal cortex, area 13, brain region is considered. This region is involved in reward and subjective pleasure (Grabenhorst and Rolls, 2011; Rolls, 2014). The relevant voxels are in AAL2 regions such as OFCmed, OFCant, OFCpost, Olfactory and Rectus (Figures 1 and 2; Table 2). The voxels within this area 13 cluster have high positive correlations between them, and have generally the same pattern of altered FC in depression (Cheng *et al.*, 2016), so this cluster is described in the remainder of this paper as OFC13.

Second, some voxels in the amygdala have reduced FC with a more lateral to anterior part of the orbitofrontal cortex, in AAL2 areas OFClat and Frontal_Inf_Orb_2 (Figures 1 and 2), and this is an area that is BA 47/12 (Öngür *et al.*, 2003), is termed here OFC47/12, and is involved in non-reward and unpleasant events (Grabenhorst and Rolls, 2011; Rolls, 2014, 2016c). There is some overlap of the amygdala voxels with reduced connectivity in depression in the medial and lateral orbitofrontal cortex areas, but some other amygdala voxels have reduced FC only with the medial orbitofrontal cortex, perhaps because more amygdala voxels have significant FC with the more extensive medial than with the lateral orbitofrontal cortex.

Third, some voxels in the amygdala have decreased FC with some temporal cortex areas including the inferior temporal gyrus, fusiform gyrus and temporal pole, areas known to be involved in visual and multimodal processing (Rolls, 2012, 2016a) (Figures 1 and 2; Table 2). This reduced FC also extended posteriorly into earlier visual areas including the occipital cortex and lingual gyrus, areas to which the amygdala sends backprojections (Amaral *et al.*, 1992).

Fourth, some amygdala voxels had reduced FC in depression with medial temporal lobe areas such as the parahippocampal, perirhinal and entorhinal cortex implicated in memory (Figures 1 and 2; Table 2).

Fifth, some amygdala voxels were found to have reduced FC with the insula (Figures 1 and 2; Table 2), in an area implicated in autonomic output (Rolls, 2016b).

Sixth, some amygdala voxels were found to have reduced FC with more ventral parts of the striatum including parts of the caudate and putamen (Figures 1 and 2; Table 2).

Seventh, some amygdala voxels were found to have reduced FC with some somatosensory/motor areas including the middle cingulate cortex and pre- and post-central gyrus (Figures 1 and 2; Table 2).

### Amygdala voxel-level FC in healthy participants, using parcellation

**Fig. 3.**
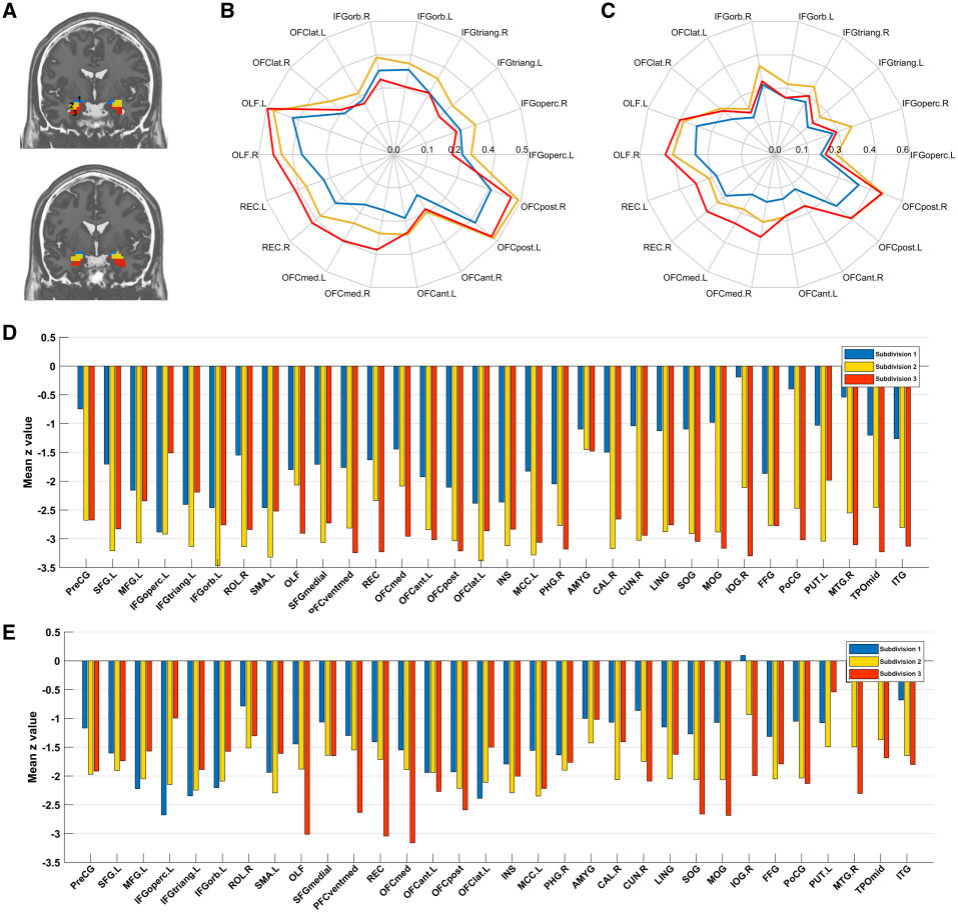
Voxel-level parcellation of the amygdala. Voxel-level parcellation in controls. (A) The three subdivisions shown on coronal slices at *Y*=−4 (top) and *Y*=−1. The left side of the brain is on the left of the images. (B) Voxel-level parcellation of the left amygdala (AMYG) based on its FC in healthy controls with other brain areas. The polar plot shows the correlations of the voxels in each subdivision of the amygdala with the significantly different voxels in orbitofrontal cortex AAL2 areas. FC (*r*) values are indicated by the distance from the centre of the polar plot, with the scale shown indicating the *r* value. A two-way repeated measures analysis of variance (ANOVA) showed by the interaction term (*P* < 0.0001) that the three amygdala subdivisions had different FC with these orbitofrontal cortex areas. (C) Voxel-level parcellation of the right amygdala based on its FC in healthy controls with other brain areas. The polar plot shows the correlations of the voxels in each subdivision of the AMYG with the significantly different voxels in orbitofrontal cortex AAL2 areas. The interaction term in the ANOVA was again significant. Differences in FC for these three divisions of the amygdala in depression. (D) The mean *z* value for the difference in FC (patients with depression—healthy controls) of the links between voxels in each subdivision and the significant ROIs showed in Table 2 for the left AMYG. (E) The same as (D) for the right AMYG.

To analyse whether some parts of the amygdala had changes especially related to depression, we first performed a parcellation of the amygdala in healthy controls, and then examined the differences of FC of each division of the amygdala between controls and participants with depression. The parcellation analysis was performed on the healthy controls, so that we could investigate how the strengths of the connectivities in different parts of the amygdala in healthy individuals might be different in depression. For healthy control participants the voxel-wise FC pattern of the amygdala with voxels in other brain areas is shown in Figure 3A–C (*k*-means was used to perform this clustering. The number of clusters was selected to be the maximum number in which each cluster was spatially discrete). Three subdivisions were found in each hemisphere (Figure 3A), a dorsal amygdala subdivision close to the central nucleus of the amygdala (1, blue); a dorsolateral amygdala subdivision (2, yellow), and a ventral amygdala subdivision (3, red). Similar parcellation was found, if the FC of each amygdala voxel with other amygdala voxels was used to perform the clustering. The pattern of FC for the different amygdala subdivisions with areas of the orbitofrontal cortex is different, as shown in the diagrams in Figure 3B and C. The basolateral part of the amygdala (subdivision 3, red) has high FC with medial orbitofrontal cortex areas (including AAL2 areas REC and OFCmed). The dorsolateral part of the amygdala (subdivision 2, yellow) has especially strong FC with the lateral orbitofrontal cortex and its related ventral parts of the inferior frontal gyrus (including AAL2 areas OFClat, IFGorb, IFGtriang and IFG operc). The dorsal part of the amygdala (subdivision 1, blue) has relatively strong FC with the posterior orbitofrontal cortex (AAL2 area OFCpost and the area immediately posterior to this, OLF which includes the olfactory tubercle).

### Functional connectivities for different amygdala subregions in depression

The differences of FC in depression of these three amygdala subdivisions are shown in Figure 3D and E. The contrast is (healthy controls—depressed group) for the three subdivisions. (A negative value for z in Figure 3D and E thus represents a weaker FC in patients with depression.) Figure 3D and E shows that all three amygdala subdivisions show reduced FC with the AAL2 areas listed in Table 2. A difference that was consistent across both hemispheres is that the basal amygdala division (subdivision 3, red) had especially reduced FC with some medial orbitofrontal cortex areas (including AAL2 areas OFCmed, REC, PFCventmed and OLF). In contrast, the dorsolateral amygdala subdivision (2, yellow) had relatively reduced FC with lateral orbitofrontal cortex and related areas (including AAL2 areas OFClat, IFGorb, IFGtriang and IFGoperc) (relative to subdivision 3). These differences are of interest, for the medial orbitofrontal cortex is involved in reward, and the lateral orbitofrontal cortex in non-reward and punishment (Grabenhorst and Rolls, 2011; Rolls, 2014, 2017b).

### Clinical symptom correlates of the reduced amygdala functional connectivities in depression

The hypothesis that some of the symptom scores or the illness duration was related to the reduced FC identified between the medial orbitofrontal cortex and amygdala in depression was tested by grouping together all the medial orbitofrontal cortex areas. The results (Table 3, top row) show that the reduction in FC between right amygdala voxels, and the medial orbitofrontal cortex areas (OFCmed, OFCpost, OFCant, rectus and OLF) were significantly correlated with the BDI score; and that the reduction of FC between both sides of the amygdala and the medial orbitofrontal cortex areas was related to the duration of the illness. These correlations of the FC of amygdala voxels with the medial orbitofrontal cortex areas are significant FDR corrected for multiple comparisons.

**Table 3.** Correlations between the FC links and the depression symptom severity scores

Regions	BDI score				Illness duration			
	Left Amygdala		Right Amygdala		Left Amygdala		Right Amygdala	
	*r* value	*P***value**	*r* value	*P***value**	*r* value	*P***value**	*r* value	*P***value**
**Medial OFC (FDR)**	−0.053	0.0624	−0.061	**0.0354**	−0.0735	0.0180*	−0.0846	0.0072*
Precentral_R	−0.075	**0.0420**	−0.087	**0.0190**	−0.039	0.1814	−0.036	0.2018
Frontal_Sup_2_L	−0.036	0.1450	−0.028	0.2160	−0.065	**0.0346**	−0.072	**0.0216**
Rolandic_Oper_R	−0.059	0.0950	−0.086	**0.0330**	−0.033	0.2122	−0.040	0.1744
Olfactory_L	−0.023	0.3120	−0.049	0.1300	−0.117	**0.0028**	−0.115	**0.0024**
Olfactory_R	−0.062	**0.0440**	−0.092	**0.0030**	−0.093	**0.0056**	−0.068	**0.0314**
Frontal_Sup_Medial_L	−0.048	0.0950	−0.057	0.0790	−0.067	**0.0436**	−0.062	0.0580
Frontal_Sup_Medial_R	−0.093	**0.0140**	−0.104	**0.0060**	−0.068	0.0616	−0.013	0.3890
Frontal_Med_Orb_L	−0.033	0.2230	−0.041	0.1730	−0.080	**0.0346**	−0.087	**0.0270**
Rectus_L	−0.031	0.2410	−0.049	0.1270	−0.066	0.0640	−0.104	**0.0088**
Rectus_R	−0.030	0.2450	−0.064	0.0560	−0.082	**0.0252**	−0.090	**0.0146**
OFCmed_L	−0.052	0.1170	−0.060	0.0660	−0.054	0.1006	−0.101	**0.0072**
OFCmed_R	−0.059	0.0640	−0.080	**0.0190**	−0.057	0.0800	−0.073	**0.0316**
OFCant_L	−0.035	0.1860	−0.041	0.1260	−0.058	0.0628	−0.070	**0.0312**
OFCpost_L	−0.068	**0.0340**	−0.049	0.0970	−0.078	**0.0208**	−0.085	**0.0090**
OFCpost_R	−0.080	**0.0100**	−0.071	**0.0280**	−0.089	**0.0078**	−0.053	0.0856
Insula_L	−0.067	**0.0460**	−0.072	**0.0460**	−0.095	**0.0112**	−0.089	**0.0210**
Insula_R	−0.056	0.0560	−0.071	**0.0290**	−0.045	0.1016	−0.029	0.2142
Cingulate_Mid_L	−0.080	**0.0340**	−0.099	**0.0130**	−0.029	0.2568	−0.051	0.1320
Amygdala_L	−0.055	0.0750	−0.063	**0.0450**	−0.049	0.0952	−0.027	0.2450
Amygdala_R	−0.064	**0.0440**	−0.081	**0.0160**	−0.026	0.2544	−0.034	0.1908
Calcarine_R	−0.054	0.1220	−0.124	**0.0050**	−0.029	0.2586	−0.041	0.1724
Cuneus_R	−0.028	0.2710	−0.125	**0.0050**	−0.044	0.1730	−0.028	0.2682
Lingual_L	−0.059	0.1010	−0.088	**0.0250**	−0.004	0.4622	−0.012	0.3924
Lingual_R	−0.038	0.2040	−0.090	**0.0340**	−0.002	0.4768	−0.034	0.2200
Occipital_Sup_L	−0.022	0.3030	−0.082	**0.0380**	−0.053	0.1212	−0.052	0.1292
Occipital_Sup_R	−0.004	0.4480	−0.089	**0.0250**	−0.059	0.0960	−0.041	0.1840
Occipital_Mid_L	−0.031	0.2190	−0.085	**0.0220**	−0.049	0.1230	−0.033	0.2324
Occipital_Mid_R	−0.027	0.2550	−0.086	**0.0250**	−0.082	**0.0270**	−0.085	**0.0318**
Occipital_Inf_R	−0.062	0.0860	−0.096	**0.0140**	−0.033	0.2364	−0.051	0.1242
Postcentral_L	−0.067	0.0740	−0.091	**0.0270**	−0.024	0.3036	−0.020	0.3260
Putamen_L	−0.053	0.1240	−0.104	**0.0090**	−0.027	0.2848	0.021	0.6726
Temporal_Mid_R	−0.084	**0.0260**	−0.114	**0.0040**	−0.012	0.3846	−0.022	0.3114
Temporal_Pole_Mid_L	−0.047	0.1130	−0.035	0.2030	−0.053	0.1016	−0.076	**0.0354**
Temporal_Pole_Mid_R	−0.054	0.0730	−0.043	0.1320	−0.080	**0.0202**	−0.065	0.0528
Temporal_Inf_R	−0.077	**0.0210**	−0.087	**0.0160**	−0.037	0.1614	−0.026	0.2554

In the top row of the table, the correlations are shown for the average of all FDR significant amygdala voxels with the average of all FDR significant medial orbitofrontal voxels from the medial OFC AAL2 areas, and these statistics are significant when considering the correlations with the symptoms of just the amygdala and medial orbitofrontal cortex voxels (FDR, *P* < 0.05). The rest of the table provides, for illustrative purposes, correlations of the symptoms with the average strength of the FC between significant voxels in the amygdala with those in other AAL2 brain areas. The values in the lower part of the Table in bold font are significant at *P* < 0.05 (permutation test, uncorrected).

* Significant *P* < 0.05 after FDR correction. Supplementary Figure S1 shows for illustrative purposes the locations of the voxels in the amygdala in brain slices that have significant correlations (*P* < 0.05) with the BDI scores or with illness duration.

In the remainder of Table 3 and in Supplementary Figure S1, we show for illustration that for individual AAL2 areas there were significant correlations (*P* < 0.05 uncorrected) between some of the AAL2-based region of interest-wise FC links and the symptom severity scores and illness duration. The correlations are in the direction that the weaker an FC link is, the higher is the clinical score for the depression. For Table 3, the correlation is that between the average of the FC of the FDR corrected significant voxels in an AAL2 region with an amygdala voxel, and the clinical measures. The BDI score was correlated with decreased FC between the amygdala and the OFC_med, and OFC_post, with the cuneus, with the fusiform cortex, with the temporal pole, with the mid-cingulate cortex and with occipital areas and the lingual gyrus (Table 3). The illness duration was correlated with decreased FC between the amygdala and the medial orbitofrontal cortex areas including OFCmed, OFCpost, OFCant and Gyrus Rectus; with the fusiform gyrus; and with the inferior temporal gyrus. For Supplementary Figure S1, the correlations shown are those between every voxel-based link between all voxels with FDR corrected differences in depression between the amygdala and an AAL2 region, and the clinical measures. The statistical significance of these effects is shown in Table 3. Supplementary Figure S1 shows, extremely interestingly, that a decrease of FCs of the medial orbitofrontal cortex with the amygdala is correlated with more severe depression (measured by the BDI and illness duration). To address any possible effect of the medication on the correlation between the clinical variables and functional connectivities, we also performed a partial correlation as described in the section ‘Methods’ by adding medication, head motion, education, sex and age as covariates. The results were consistent with the correlations shown in Table 3, which indicates that the results in Table 3 hold independently of the medication. The HAMD (Hamilton, 1960) score was not significantly correlated with the functional connectivities of the amygdala.

### FC in unmedicated patients with depression

Within the depressed group from the Xinan dataset, 125 were not receiving medication, and 157 patients were receiving medication. We were able to confirm that the main findings shown here in Figure 1 for all the depressed patients were not due just to the effects of the medication, in that in the 125 unmedicated patients, differences from controls in the same brain regions were similar, as shown in Supplementary Figure S2. However, it was of interest that in the unmedicated patients, the precuneus had higher FC with the amygdala (see Supplementary Figure S2). Consistently, in further analyses, in which the unmedicated and medicated depressed patients were compared, it was found that in the medicated patients there was much less of an increase of FC between the precuneus and the amygdala. It thus may be that the medication decreases the FC between the precuneus and amygdala. (The medication consisted in most cases of selective serotonin reuptake inhibitors including fluoxetine, paroxetine, sertraline, citalopram and escitalopram; or serotonin-norepinephrine reuptake inhibitors such as venflaxine, or a tetracyclic antidepressant such as mirtazepine.) In this study, any effects of the medication were difficult to analyse, because those on medication were more likely to be long-term than first episode patients, so we limit the analysis to what has just been described. In summary, the reductions in FC shown in Figure 1 and Table 2 in patients with depression were also found in unmedicated patients (Supplementary Figure S2), and are not due just to the effects of the medication.

## Discussion

**Fig. 4.**
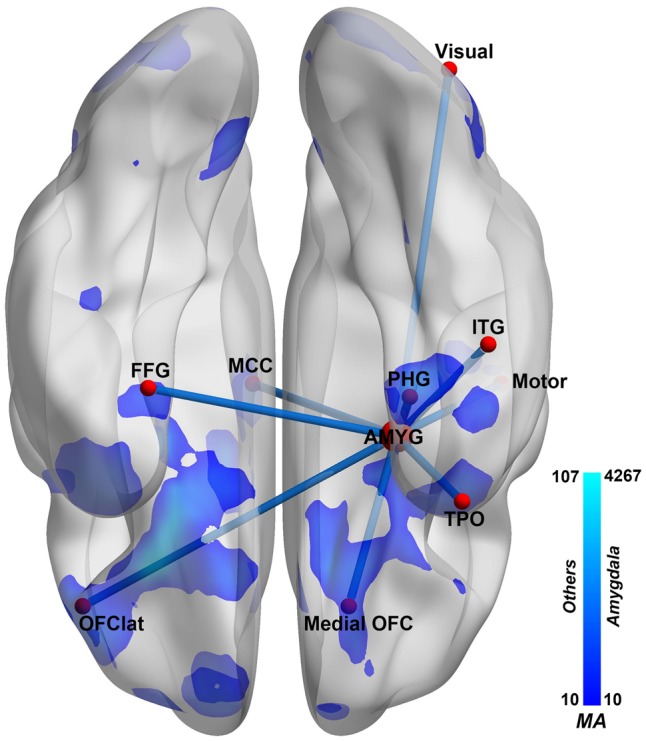
Summary of amygdala FC differences in depression. The amygdala networks that show different FC in patients with depression. Left: ventral view. Ventral view of the brain. A decrease in FC is shown in blue, and an increase in red, at the voxel level, with the scale shown on the right calibrated using the measure of Association of each voxel (MA, see text). AMYG, amygdala; HIP, hippocampus; ITG, inferior temporal gyrus; MCC, mid-cingulate cortex; motor, pre- and post-central gyrus and Rolandic operculum; OFC, orbitofrontal cortex; PHG, parahippocampal area; FFG, fusiform gyrus; TPO, temporal pole; visual, some occipital areas (see Table 1). Voxels with different FC from controls are shown by the blue shading, with decreases evident in depression.

One main finding is that the amygdala has reduced FC with a major region with altered FC in depression, the medial orbitofrontal cortex BA 13 (Cheng *et al.*, 2016), which is implicated in reward (Grabenhorst and Rolls, 2011; Rolls, 2014) (Table 2; Figures 1–4). The reduced FC of the amygdala with the medial orbitofrontal cortex was correlated with the increase in the measures from the BDI (Table 3; Supplementary Figure S1), and with the illness duration, making it likely that this FC link between the amygdala and the medial orbitofrontal cortex is related to the depression. This finding is consistent with the non-reward attractor theory of depression, which includes the hypothesis of less activity in medial orbitofrontal cortex reward-related areas in depression (Rolls, 2016c), as well as increased activity in the reciprocally related lateral orbitofrontal cortex. The new theory is that the lateral orbitofrontal frontal cortex, which is activated when expected reward is not obtained (which can cause sadness), can enter and maintain an ongoing mood state in a recurrent ‘attractor’ network more readily in depression (Rolls, 2016c), This attractor or short-term memory state may be triggered into increased activity by a strong non-reward event in the environment, or may be more sensitive in some depressed people, and more easily therefore triggered into a high firing rate state that is associated with a sad mood. Given that the amygdala has some roles in emotion (Aggleton, 2000; Whalen and Phelps, 2009; LeDoux, 2012; Rolls, 2014), its reduced FC with the medial orbitofrontal cortex which is involved in reward and positive mood, may contribute to the lowering of mood by being somewhat disconnected from the orbitofrontal cortex in depression. A possible clinical implication is that altering the functioning of the lateral orbitofrontal cortex may release the medial orbitofrontal cortex. There is already evidence that repetitive transcranial stimulation (rTMS) of the lateral orbitofrontal cortex may be helpful in the treatment of some patients with depression (Fettes *et al.*, 2017; Feffer *et al.*, 2018).

An additional part of the theory of depression is that, given that the lateral and medial orbitofrontal cortex tend to have reciprocal activations (O'Doherty *et al.*, 2001; Rolls, 2014), decreased activity of the reward-related medial orbitofrontal cortex may also be related to depression (Rolls, 2016c, 2017b, 2018). Consistent with this, the medial orbitofrontal cortex has reduced FC with systems implicated in memory, including the medial temporal lobe (Cheng *et al.*, 2016), and posterior cingulate cortex (Cheng *et al.*, 2018a) [which can be considered a gateway to the hippocampus (Rolls, 2017d; Rolls and Wirth, 2018)]. This reduced FC may reduce happy memories. It is suggested in this context that the reduced FC in depression of the amygdala with the medial orbitofrontal cortex described here is related to the reduced hedonia in depression.

It is noted that the great development of the orbitofrontal cortex in primates and especially in humans in evolution (Rolls, 2017a), may enable the human orbitofrontal cortex to make a correspondingly greater contribution to emotion in humans than the amygdala (Rolls, 2014, 2017a), and to depression. Indeed, the FC changes of the medial and lateral orbitofrontal cortex were significant for large numbers of voxels in the whole brain FC analysis (Cheng *et al.*, 2016).

Second, some FC reductions of the amygdala with medial temporal lobe areas such as the parahippocampal, perirhinal and entorhinal cortex implicated in memory were found (Figures 1–4; Table 2). This decrease in FC of the amygdala with medial temporal lobe memory-related areas is similar to that of the medial orbitofrontal cortex, which has greatly reduced FC with the medial temporal lobe memory system (Cheng *et al.*, 2016), and which with its role in reward may be related to the reduced processing of happy memories, and therefore an imbalance towards unhappy memories, in depression (Cheng *et al.*, 2016; Rolls, 2016c).

Third, some voxels in the amygdala had decreased FC with some temporal cortex areas including the inferior temporal gyrus, temporal pole, and fusiform gyrus, areas known to be involved in visual and multimodal processing (Rolls, 2012, 2016a) (Figures 1 and 2; Table 2). These decreases of these functional connectivities were correlated with the severity of the symptoms and the illness duration (Table 3; Supplementary Figure S1), and these functional connectivities were higher in medicated than unmedicated patients (Supplementary Figure S2). This is thus strong evidence that the reduction of connectivity between temporal cortex areas and the amygdala is important in depression. These temporal cortex areas may introduce inputs relevant to emotion to the amygdala, in that neurons in the primate inferior temporal visual cortex respond to faces (Perrett *et al.*, 1982; Rolls, 2011, 2012), and similar neurons are found in the amygdala (Leonard *et al.*, 1985), linking these regions to emotional responses to faces. The hypothesis is that these temporal cortical areas provide important inputs to the amygdala (Rolls, 2014), and that backprojections from the amygdala reach these areas and also earlier cortical including occipital visual areas (Amaral and Price, 1984; Amaral *et al.*, 1992; Rolls, 2016a).

Fourth, some amygdala voxels had reduced FC with the middle cingulate cortex, involved in motor function, in depression. This pathway has been identified in macaques, and it has been suggested is involved in influences of amygdala face processing subsystems (Rolls, 2007, 2011) on emotional face expressions associated with social communication and emotional constructs such as fear, anger, happiness and sadness (Morecraft *et al.*, 2007). Interestingly, no effects were found relating the amygdala in depression to a different cingulate area involved in reward and pleasure, the anterior cingulate cortex (Grabenhorst and Rolls, 2011; Rolls, 2014). This again emphasizes the importance of the orbitofrontal cortex and the regions connected to it in depression (Rolls, 2016c).

Fifth, the results of the parcellation of the amygdala based on its voxel-level FC in 350 healthy controls are of great interest. The basal division of the amygdala (subdivision 3, red in Figure 3) has high FC with medial orbitofrontal cortex areas, and the dorsolateral part of the amygdala (subdivision 2, yellow) has especially strong FC with the lateral orbitofrontal cortex and its related ventral parts of the inferior frontal gyrus. In depression, the basal amygdala division had especially reduced FC with medial orbitofrontal cortex areas; and the dorsolateral amygdala subdivision (2, yellow) had relatively reduced FC with lateral orbitofrontal cortex and related areas. These differences are of interest, for the medial orbitofrontal cortex is involved in reward, and the lateral orbitofrontal cortex in non-reward and punishment (Grabenhorst and Rolls, 2011; Rolls, 2014, 2017b). At the cytoarchitectonic level, three main divisions have been described (Amunts *et al.*, 2005), a centromedial group (the central nucleus and medial nucleus) which may correspond to our subdivision 1; a superficial group (which may correspond to our functional subdivision 2); and a laterobasal group (which may correspond to our functional subdivision 3). However, as a cytoarchitectonic study, that did not reveal evidence about the connectivity of the three subdivisions of the amygdala, which is provided by the present results.

A strength of this study is the large number of participants, which enabled robust voxel-level FC to be analysed, enabling identification of precisely defined parts of brain areas that had altered connectivity that was related to the depression, such as the lateral part of the lateral orbitofrontal cortex. A limitation is that it would be useful to extend this investigation to activations of the amygdala; and also to effective (that is directed) connectivity (Gilson *et al.*, 2016). From the present investigation, it appears that the main routes via which the amygdala has altered connectivity with other brain regions that may contribute to depression are via the medial orbitofrontal cortex, the lateral orbitofrontal cortex, the medial temporal lobe memory-related areas, and the temporal cortex. Another possible route is via the basal ganglia (see Table 2), which have connections with the habenula, which in turn provides a route for cortical areas and the amygdala to influence serotonergic and dopaminergic neurons (Rolls, 2017c). The relatively few differences in the amygdala FC in depression with some other areas implicated in depression, including the anterior and subcallosal cingulate cortex (Mayberg, 2003; Hamani *et al.*, 2011; Laxton *et al.*, 2013; Lujan *et al.*, 2013) was notable, and in contrast to the orbitofrontal cortex (Cheng *et al.*, 2016; Rolls, 2016a,c). Moreover, and consistently, the changes of FC of the amygdala in depression were less significant than the changes in other areas such as the medial orbitofrontal cortex, lateral orbitofrontal cortex and parahippocampal gyrus (Cheng *et al.*, 2016). A possible limitation of the study was that acutely depressed and remitted depressed patients were included, and there might be differences. Further, although some of the patients were on medication, a highlight of the study was that we were able to examine the differences in a much larger group of unmedicated patients than has ever been studied previously. The importance of the present study is that by focusing on the amygdala, and using very large neuroimaging datasets of patients with depression and controls, we were able to characterize the altered FC in depression of the amygdala with other brain regions.

## Supplementary data

Supplementary data are available at *SCAN* online.

## Authors’ Contributions

Wei Cheng, Edmund T. Rolls and Jianfeng Feng contributed to the design of the study. Jiang Qiu, Xiongfei Xie, Hongtao Ruan, Yu Li, Chu-Chung Huang, Albert C. Yang, Shih-Jen Tsai, Fajin Lv, Kaixiang Zhuang, Ching-Po Lin and Peng Xie contributed to the collection of the data. Wei Cheng, Edmund T. Rolls and Wujun Lv contributed to the analysis of the data and the preparation of the manuscript. Edmund T. Rolls, Wei Cheng and Jianfeng Feng participated in writing the paper. All collaborators had an opportunity to contribute to the interpretation of the results and to the drafting of the manuscript.

## Funding

J.F. is partially supported by the key project of Shanghai Science & Technology Innovation Plan (Nos. 15JC1400101 and 16JC1420402) and the National Natural Science Foundation of China (Grant Nos. 71661167002 and 91630314). The research was also partially supported by the Shanghai AI Platform for Diagnosis and Treatment of Brain Diseases (No. 2016-17). The research was also partially supported by Base for Introducing Talents of Discipline to Universities No. B18015. W.C. is supported by grants from the National Natural Sciences Foundation of China (Nos. 81701773, 11771010), Sponsored by Shanghai Sailing Program (No. 17YF1426200) and the Research Fund for the Doctoral Program of Higher Education of China (No. 2017M610226). W.C. is also sponsored by Natural Science Foundation of Shanghai (No. 18ZR1404400). C.P.L. was supported in part by funding from Ministry of Science and Technology, Taiwan (NSC100-2911-I-010-010, NSC101-2911-I-010-009, NSC100-2628-E-010-002-MY3, NSC102-2321-B-010-023 and NSC103-2911-I-010-501), National Health Research Institutes (NHRI-EX103-10310EI), Ministry of Health and Welfare of Taiwan (DOH102-TD-PB-111-NSC006), and Academia Sinica, Taipei, Taiwan. J.Q. was supported by the National Natural Science Foundation of China (31271087, 31470981, 31571137, 31500885), National Outstanding young people plan, the Program for the Top Young Talents by Chongqing, the Fundamental Research Funds for the Central Universities (SWU1509383), Natural Science Foundation of Chongqing (cstc2015jcyjA10106), General Financial Grant from the China Postdoctoral Science Foundation (2015M572423). P.X. is supported by National Key R&D Program of China (2017YFA0505700). 

*Conflict of interest*. None declared.

## Supplementary Material

Supplementary Data
