# Supplementary material for: Functional connectivity of the human amygdala in health and in depression
Source: Soc Cogn Affect Neurosci. 2018 May 14;13(6):557–68. doi: 10.1093/scan/nsy032 (PMC6022538; doi:10.1093/scan/nsy032)
Supplement: Supplementary Data [file nsy032_scan-17-443-file006.docx]

**Functional connectivity of the human amygdala in health and in depression**

**Supplementary Material**

Wei Cheng^1, #^; Edmund T. Rolls^2,3, #^; Jiang Qiu^4,5, #^; Xiongfei Xie^14,#^; Wujun Lyu^13^; Yu Li^5^; Chu-Chung Huang^7^; Albert C. Yang^11^; Shih-Jen Tsai^11^; Fajin Lyu^14^; Kaixiang Zhuang^5^; Ching-Po Lin^1,6,7,*^; Peng Xie^8,9,10,*^; Jianfeng Feng^1, 2, 12,*^

1. Institute of Science and Technology for Brain-Inspired Intelligence, Fudan University, Shanghai, 200433, China

2. Department of Computer Science, University of Warwick, Coventry CV4 7AL, UK

3. Oxford Centre for Computational Neuroscience, Oxford, UK

4. Key Laboratory of Cognition and Personality (SWU), Ministry of Education, Chongqing, China

5. Department of Psychology, Southwest University, Chongqing, China

6. Brain Research Center, National Yang-Ming University, Taipei, Taiwan

7. Institute of Neuroscience, National Yang-Ming University, Taipei, Taiwan

8. Institute of Neuroscience, Chongqing Medical University, Chongqing, China

9. Chongqing Key Laboratory of Neurobiology, Chongqing, China

10. Department of Neurology, The First Affiliated Hospital of Chongqing Medical University, Chongqing, China

11. Department of Psychiatry, Taipei Veterans General Hospital, Taipei, Taiwan

12. School of Mathematical Sciences, School of Life Science and the Collaborative Innovation Center for Brain Science, Fudan University, Shanghai, 200433, PR China

13. School of Mathematics, Shanghai University Finance and Economics, Shanghai, 200433, PR China

14. Department of Radiology, The First Affiliated Hospital of Chongqing Medical University, Chongqing, China

^#^ These authors contributed equally to this work.

**Social, Cognitive, and Affective Neuroscience (2018)**

**Supplementary Methods**

**Participants**

There were 336 patients with a diagnosis of major depressive disorder (MDD), and 350 controls. The patients were from Taiwan and Xinan. 125 of the patients were not receiving medication at the time of the neuroimaging. The subjects' consent was obtained according to the Declaration of Helsinki and the investigations were approved by the ethical committee of the institution in which the work was performed. Further details follow.

**Xinan:** Patients with MDD were recruited from the outpatient department of the First Affiliated Hospital of Chongqing Medical School in Chongqing, China. All were diagnosed according to the Structured Clinical Interview for DSM-IV, by independent assessments of two psychiatrists. They were also assessed for disease severity using the Hamilton Depression Rating Scale (HAMD) (Hamilton, 1960) and Beck Depression Inventory (BDI), illness duration and the medication status of the patients. Before the investigation, we excluded individuals who were not suitable for MRI scanning by interview and by the self-reported checklist. The MRI related exclusion criteria include claustrophobia, metallic implants, Meniere’s Syndrome and a history of fainting within the previous half year. Exclusion criteria for both groups were as follows: current psychiatric disorders (except for MDD) and neurological disorders; substance abuse; and stroke or serious encephalopathy. Of note, all of the subjects in the control group did not meet DSM-IV criteria for any psychiatric disorders and did not use any drugs that could affect brain function. This study was approved by the Research Ethics Committee of the Brain Imaging Center of Southwest University and First Affiliated Hospital of Chongqing Medical School. Informed written consent was obtained from each subject. This study was conducted in accordance with the Helsinki Declaration as revised in 1989.

**Taiwan:** Patients were recruited from the Veteran General Hospital in Taipei, Taiwan. All participants were diagnosed according to the Diagnostic and Statistical Manual of Mental Disorder-IV criteria for depression, and each participant's history of medical disease, psychiatric illness, and medication use was evaluated by interview and medical charts carefully. Experiments were conducted in accordance with the Declaration of Helsinki and approved by the Institutional Review Board of Taipei Veterans General Hospital. Written informed consent was obtained from all participants after ensuring adequate understanding of the study. Any participants with the following conditions were excluded: (1) a comorbid substance-related disorder, (2) presence of neurobiological disorders, such as dementia, head injury, stroke, or Parkinson’s disease; (3) presence of hypertension, diabetes, hyperlipidemia or coronary heart disease; (4) severe medical illness, such as malignancy, heart failure, or renal failure; (4) presence of ferromagnetic foreign bodies or implants that were anywhere in the body. Depression severity was evaluated by the psychiatrist-assessed Hamilton Depression Rating Scale (HAMD, 17 items) (Hamilton, 1960).

**Image Acquisition**

Data for resting state functional connectivity analysis were collected in 3T MRI scanners in an 8 min period in which the participants were awake in the scanner not performing a task using standard protocols described in more detail next.

**Xinan:** All images were acquired on a 3.0-T Siemens Trio MRI scanner using a 16-channel whole-brain coil (Siemens Medical, Erlangen, Germany). High- resolution T1-weighted 3D images were acquired using a magnetization-prepared rapid gradient echo (MPRAGE) sequence (echo time (TE) = 2.52 ms; repetition time (TR) = 1900 ms; inversion time (TI) = 900 ms; flip angle = 9 degrees; slices = 176; thickness = 1.0 mm; resolution matrix = 256×256; voxel size = 1×1×1 mm3). For each participant, 242 functional images were acquired with a gradient echo type Echo Planar Imaging (EPI) sequence (echo time (TE) = 30 ms; repetition time (TR) = 2000 ms; flip angle = 90 degrees; slices = 32; slice thickness = 3.0 mm; slice gap = 1 mm; resolution matrix = 64×64; voxel size 3.4×3.4×3〖mm〗^3). During image acquisition, participants were instructed to keep their eyes closed while keeping their head as still as possible without falling asleep. All participants stayed awake during the MRI imaging as confirmed by the participants after the session.

**Taiwan:** fMRI scanning was performed at National Yang-Ming University in Taiwan using a 3.0-T Siemens MRI Scanner (Siemens Magnetom Tim Trio, Erlangen, Germany) with a 12-channel head coil. During the experiments, the participants were instructed to relax with their eyes closed, without falling asleep. After the resting state experiment, participants were asked whether they fell asleep during the resting state scan session, and participants were rescanned if they had fallen asleep during the resting state scan. T2*-weighted images with BOLD contrast were measured using a gradient echo- planar imaging (EPI) sequence (repetition time, TR: 2,500 ms, echo time, TE: 27 ms, field of view, FoV: 220 mm, flip angle: 77 degree, matrix size: 64 x 64, and voxel size: 3.44×3.44×3.40 mm). For each run, 200 EPI volume images were acquired in the anterior and posterior commissure (AC–PC) plane. High-resolution structural T1 images were acquired with three-dimensional (3D) magnetization-prepared rapid gradient- echo sequence (3D-MPRAGE; TR: 2,530 ms, TE: 3.5 ms, TI: 1,100 ms, FoV: 256 mm, and flip angle: 7 degree, 192 sagittal slices, voxel size = 1.0 mm x 1.0 mm 1.0 mm, no gap). For each participant, the whole fMRI scanning lasted about 16 min (T1: 8min, Resting: 8min).

**Fig. S1. The voxel wise correlation between the strength of the FCs and the Beck Depression Inventory score (A) and illness duration (B).** Only values of correlations with p < 0.05 (uncorrected) are shown.


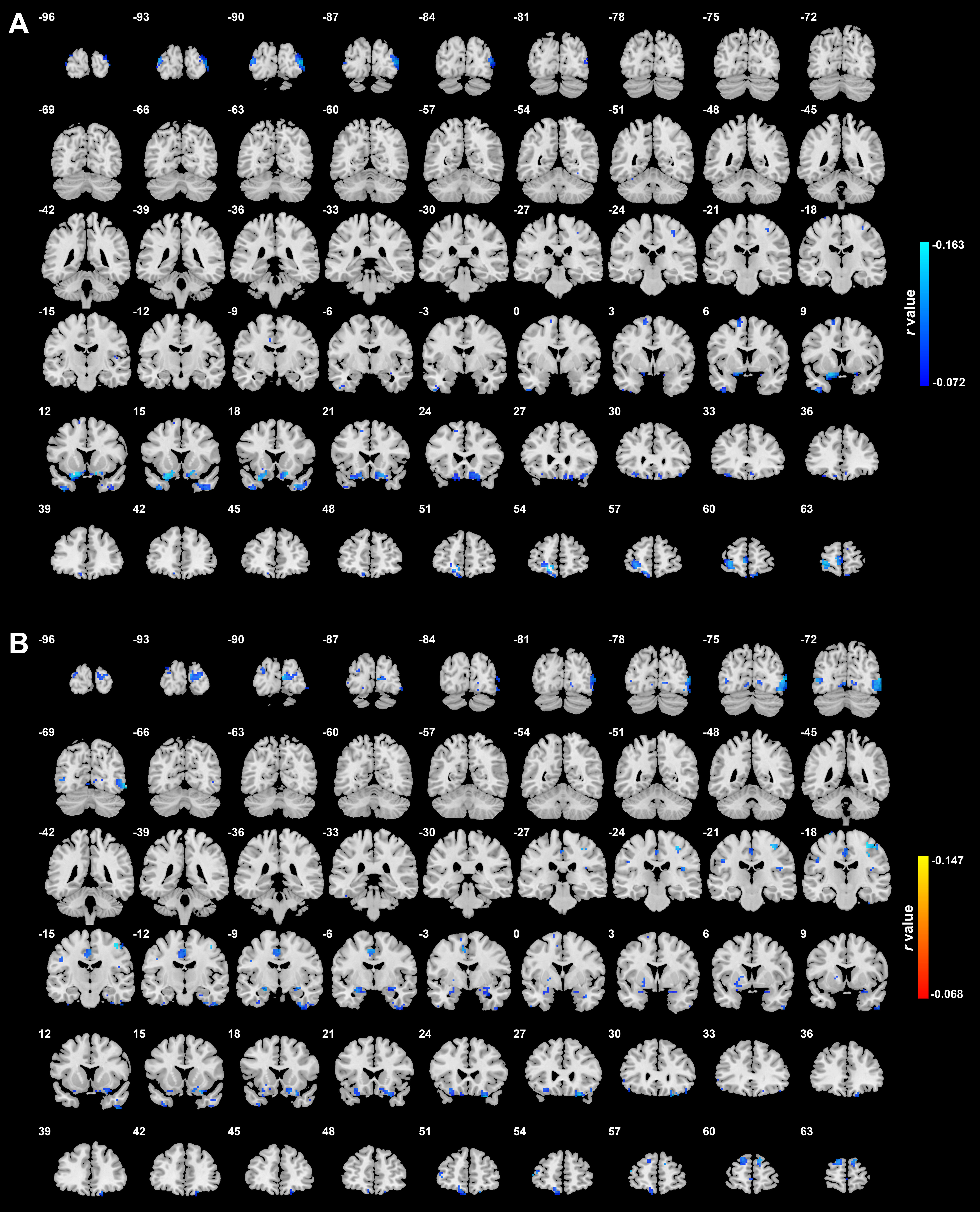


**Fig. S2. Anatomical location of consistently different functional connectivity in 125 depressed patients not receiving medication obtained from the voxel-based Association Study (vAS).** Voxels showing the largest number of voxel-level functional connectivity differences with the amygdala in patients with depression. The color bar represents the measure of association (MA) given by the number of significantly different functional connectivity links relating to each voxel. The threshold for showing functional connectivities here is the same as in the main paper Fig. 1, i.e. $6.89\times{10}^{-4}$. The right of the brain is on the right of each slice. The Y values are in MNI coordinates.


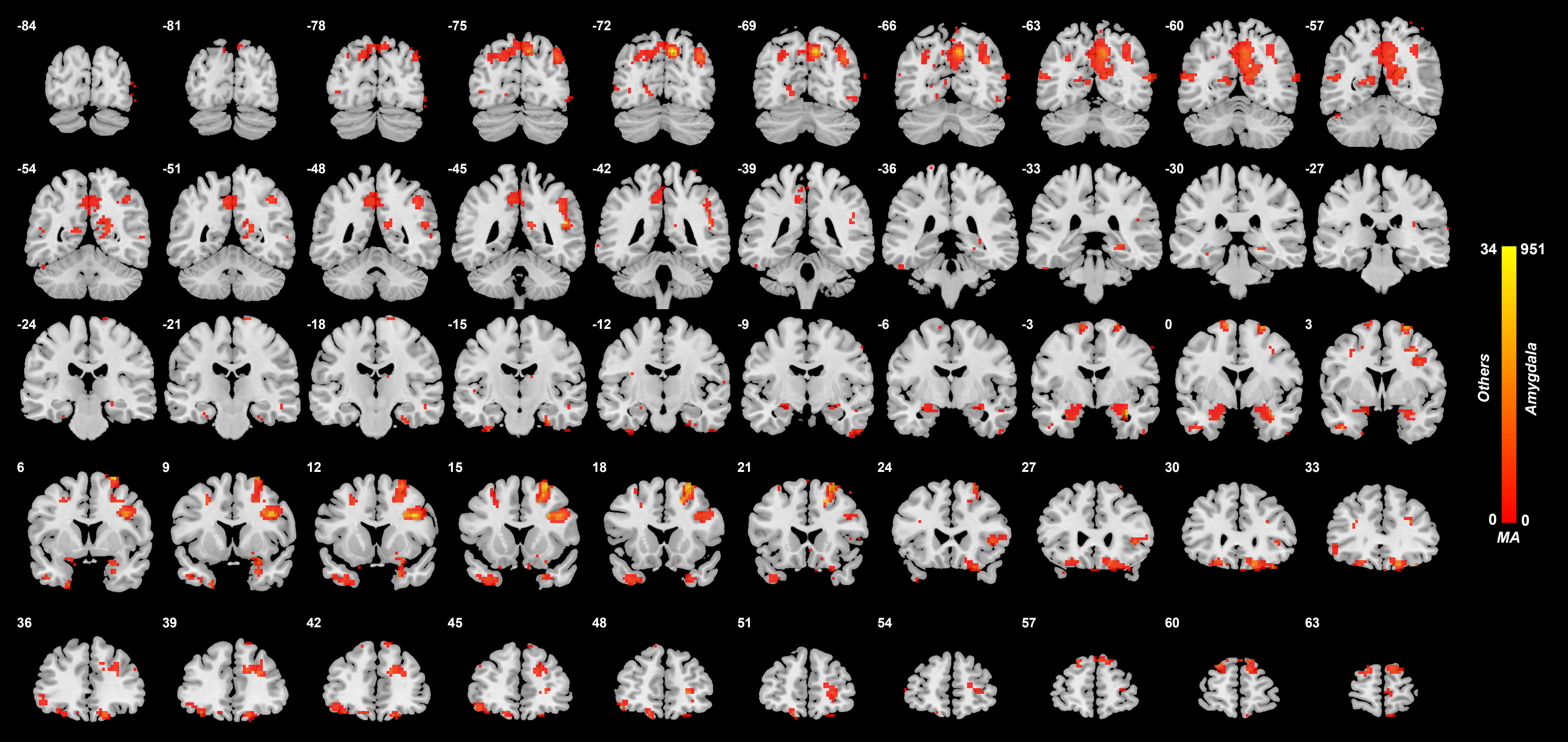


**Fig. S3. Anatomical location of consistently different functional connectivity in the Xinan dataset only obtained from the voxel-based Association Study (vAS).** This is provided to provide confirmation that the results with the combined dataset shown in Fig. 1 are consistent with those obtained in a single dataset. Voxels showing the largest number of voxel-level functional connectivity differences with the amygdala in patients with depression. The color bar represents the measure of association (MA) given by the number of significantly different functional connectivity links relating to each voxel. The threshold for showing functional connectivities here is the same as in the main paper Fig. 1, i.e. $6.89\times{10}^{-4}$. The right of the brain is on the right of each slice. The Y values are in MNI coordinates.


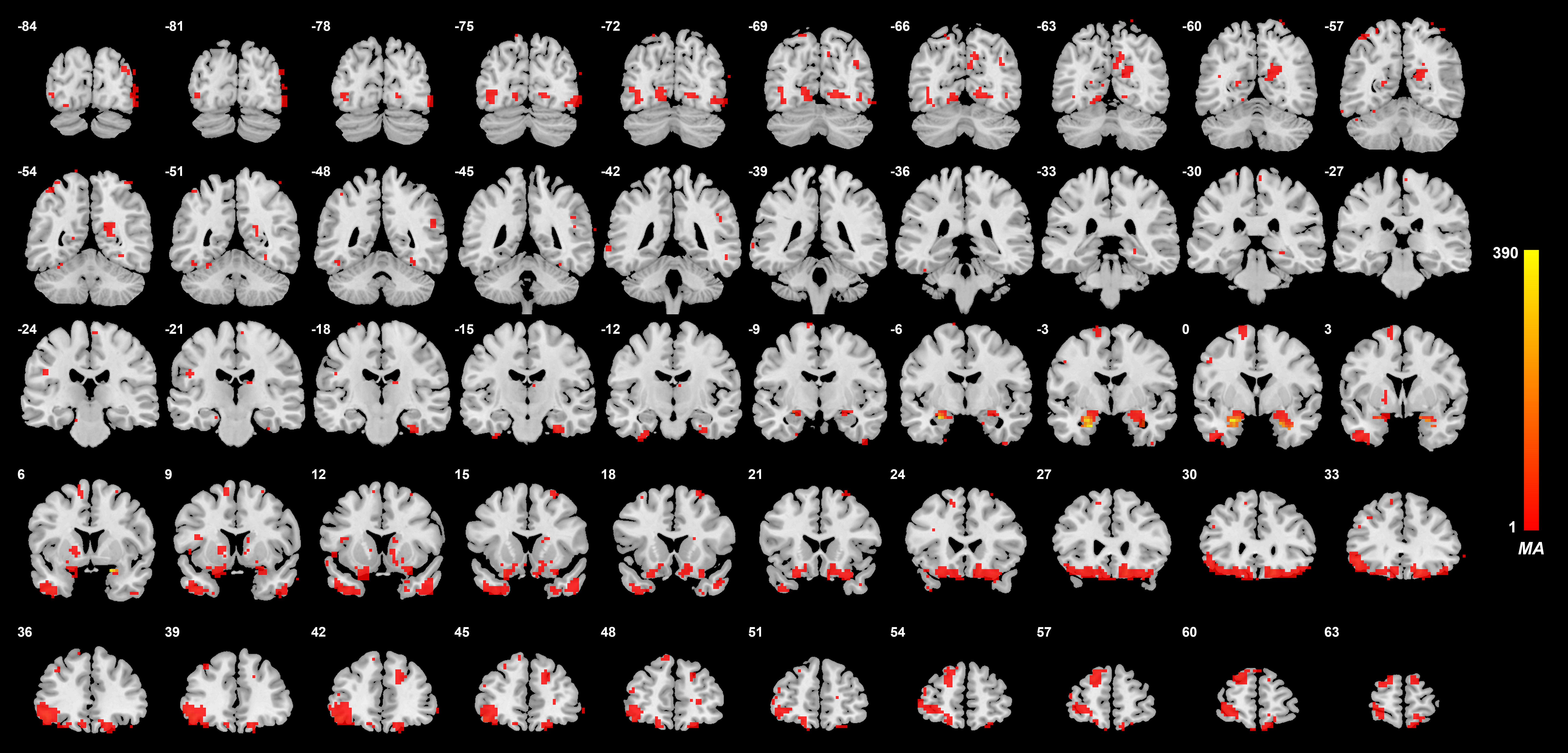


**Figure S4. A comparison of the whole brain functional connectivity difference patterns for the depressed minus the healthy group between the Xinan dataset and the Taiwan dataset.** The figure shows the correlation between the mean t value corresponding to the Xinan dataset and the mean t value corresponding to Taiwan dataset for all the voxel-wise functional connectivities involving the amygdala. This provides confirmation that the results from the two datasets are consistent.


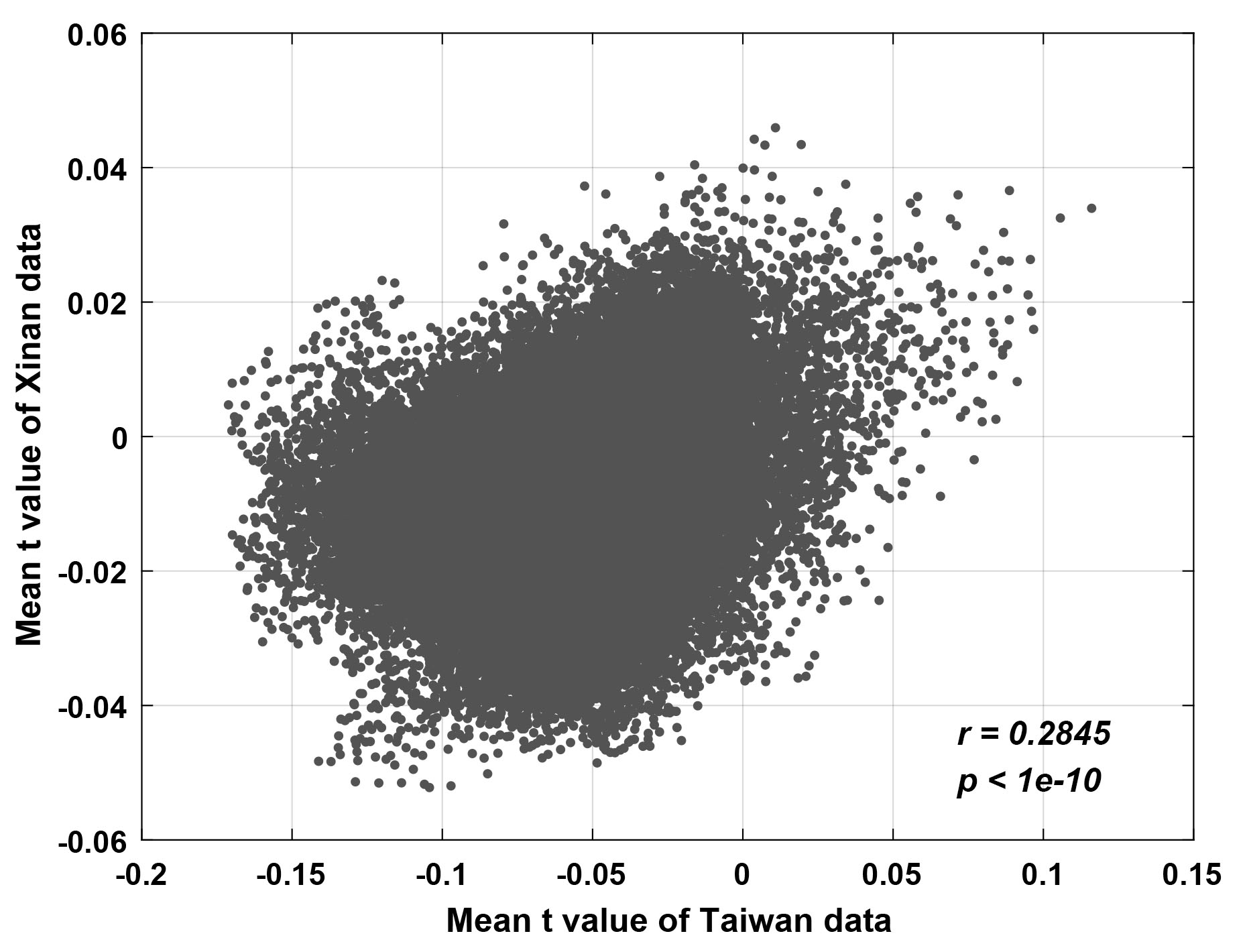


**Table S1.** The anatomical regions defined in each hemisphere and their label in the automated anatomical labelling atlas AAL2 (Rolls *et al.*, 2015). Column 4 provides a set of possible abbreviations for the anatomical descriptions.

| NO. | ANATOMICAL DESCRIPTION | LABEL  aal2.nii.gz | POSSIBLE  ABBREVIATION |
| --- | --- | --- | --- |
| 1,2 | Precentral gyrus | Precentral | PreCG |
| 3, 4 | Superior frontal gyrus, dorsolateral | Frontal_Sup | SFG |
| 5, 6 | Middle frontal gyrus | Frontal_Mid | MFG |
| 7, 8 | Inferior frontal gyrus, opercular part | Frontal_Inf_Oper | IFGoperc |
| 9, 10 | Inferior frontal gyrus, triangular part | Frontal_Inf_Tri | IFGtriang |
| 11, 12 | IFG pars orbitalis, | Frontal_Inf_Orb | IFGorb |
| 13, 14 | Rolandic operculum | Rolandic_Oper | ROL |
| 15, 16 | Supplementary motor area | Supp_Motor_Area | SMA |
| 17, 18 | Olfactory cortex | Olfactory | OLF |
| 19, 20 | Superior frontal gyrus, medial | Frontal_Sup_Med | SFGmedial |
| 21, 22 | Superior frontal gyrus, medial orbital | Frontal_Med_Orb | PFCventmed |
| 23, 24 | Gyrus rectus | Rectus | REC |
| 25, 26 | Medial orbital gyrus | OFCmed | OFCmed |
| 27, 28 | Anterior orbital gyrus | OFCant | OFCant |
| 29, 30 | Posterior orbital gyrus | OFCpost | OFCpost |
| 31, 32 | Lateral orbital gyrus | OFClat | OFClat |
| 33, 34 | Insula | Insula | INS |
| 35, 36 | Anterior cingulate & paracingulate gyri | Cingulate_Ant | ACC |
| 37, 38 | Middle cingulate & paracingulate gyri | Cingulate_Mid | MCC |
| 39, 40 | Posterior cingulate gyrus | Cingulate_Post | PCC |
| 41, 42 | Hippocampus | Hippocampus | HIP |
| 43, 44 | Parahippocampal gyrus | ParaHippocampal | PHG |
| 45, 46 | Amygdala | Amygdala | AMYG |
| 47, 48 | Calcarine fissure and surrounding cortex | Calcarine | CAL |
| 49, 50 | Cuneus | Cuneus | CUN |
| 51, 52 | Lingual gyrus | Lingual | LING |
| 53, 54 | Superior occipital gyrus | Occipital_Sup | SOG |
| 55, 56 | Middle occipital gyrus | Occipital_Mid | MOG |
| 57, 58 | Inferior occipital gyrus | Occipital_Inf | IOG |
| 59, 60 | Fusiform gyrus | Fusiform | FFG |
| 61, 62 | Postcentral gyrus | Postcentral | PoCG |
| 63, 64 | Superior parietal gyrus | Parietal_Sup | SPG |
| 65, 66 | Inferior parietal gyrus, excluding supramarginal and angular gyri | Parietal_Inf | IPG |
| 67, 68 | SupraMarginal gyrus | SupraMarginal | SMG |
| 69, 70 | Angular gyrus | Angular | ANG |
| 71, 72 | Precuneus | Precuneus | PCUN |
| 73, 74 | Paracentral lobule | Paracentral_Lobule | PCL |
| 75, 76 | Caudate nucleus | Caudate | CAU |
| 77, 78 | Lenticular nucleus, Putamen | Putamen | PUT |
| 79, 80 | Lenticular nucleus, Pallidum | Pallidum | PAL |
| 81, 82 | Thalamus | Thalamus | THA |
| 83, 84 | Heschl’s gyrus | Heschl | HES |
| 85, 86 | Superior temporal gyrus | Temporal_Sup | STG |
| 87, 88 | Temporal pole: superior temporal gyrus | Temporal_Pole_Sup | TPOsup |
| 89, 90 | Middle temporal gyrus | Temporal_Mid | MTG |
| 91, 92 | Temporal pole: middle temporal gyrus | Temporal_Pole_Mid | TPOmid |
| 93, 94 | Inferior temporal gyrus | Temporal_Inf | ITG |

References

Hamilton, M. (1960) 'A rating scale for depression', *Journal of Neurology, Neurosurgery and Psychiatry*, **23**, pp. 56-62.

Rolls, E. T., Joliot, M. & Tzourio-Mazoyer, N. (2015) 'Implementation of a new parcellation of the orbitofrontal cortex in the automated anatomical labeling atlas', *Neuroimage*, **122**, pp. 1-5.
